# Understanding Inequalities in Child Health in Ethiopia: Health Achievements Are Improving in the Period 2000–2011

**DOI:** 10.1371/journal.pone.0106460

**Published:** 2014-08-28

**Authors:** Eirin Krüger Skaftun, Merima Ali, Ole Frithjof Norheim

**Affiliations:** 1 Department of Global Public Health and Primary Care, University of Bergen, Bergen, Norway; 2 Chr. Michelsen Institute, Bergen, Norway; UCL Institute of Child Health, University College London, United Kingdom

## Abstract

**Objective:**

In Ethiopia, coverage of key health services is low, and community based services have been implemented to improve access to key services. This study aims to describe and assess the level and the distribution of health outcomes and coverage for key services in Ethiopia, and their association with socioeconomic and geographic determinants.

**Methods:**

Data were obtained from the 2000, 2005 and 2011 Ethiopian Demographic and Health Surveys. As indicators of access to health care, the following variables were included: Under-five and neonatal deaths, skilled birth attendance, coverage of vaccinations, oral rehydration therapy for diarrhoea, and antibiotics for suspected pneumonia. For each of the indicators in 2011, inequality was described by estimating their concentration index and a geographic Gini index. For further assessment of the inequalities, the concentration indices were decomposed. An index of health achievement, integrating mean coverage and the distribution of coverage, was estimated. Changes from 2000 to 2011 in coverage, inequality and health achievement were assessed.

**Results:**

Significant pro-rich inequalities were found for all indicators except treatment for suspected pneumonia in 2011. The geographic Gini index showed significant regional inequality for most indicators. The decomposition of the 2011 concentration indices revealed that the factor contributing the most to the observed inequalities was different levels of wealth. The mean of all indicators improved from 2000 to 2011, and the health achievement index improved for most indicators. The socioeconomic inequalities seem to increase from 2000 to 2011 for under-five and neonatal deaths, whereas they are stable or decreasing for the other indicators.

**Conclusion:**

There is an unequal socioeconomic and geographic distribution of health and access to key services in Ethiopia. Although the health achievement indices improved for most indicators from 2000 to 2011, socioeconomic determinants need to be addressed in order to achieve better and more fairly distributed health.

## Introduction

Evidence from low and middle income countries worldwide shows that health outcomes and access to key services are unevenly distributed across different subgroups of the population. Children from socioeconomically disadvantaged households have higher mortality rates and lower coverage of key services than children from more affluent households [1]–[4]. Geographic factors such as region of residence and distance to health facilities also influence mortality rates and coverage of health services [5]–[8]. The majority of child deaths occur from causes that are easily prevented or treated; they are therefore unnecessary and may be considered unfair [9].

Inequalities across socioeconomic groups are generally considered to be unfair, but the standardly reported measures of mean levels of health and health service coverage in the population do not tell us enough to assess the overall distribution. There is therefore a need to go beyond averages measures [10], [11].

An increase of the mean level of health and coverage may be accompanied by decreasing inequalities across the population [12], but an improvement of the mean level may also be associated with increasing inequalities [13], [14]. Policy makers may be willing to trade off equality against improvements of the mean level; a small increase of inequality may be acceptable if the mean increases, while a small increase of the mean and a large increase in inequality is not acceptable. It is therefore important to monitor health and access to key services, as well as the distribution of these in the population in order to develop and evaluate policies aimed at health and reducing inequities in health.

The per capita health expenditure in Ethiopia has increased substantially since 2000. The World Health Statistics published by the World Health Organization (WHO) estimates that the total per capita health expenditure in 2000 was 20 $ int. PPP, of which 11 were government expenditures [15]. In 2010 the total health expenditure per capita has increased to50 $ int. PPP, of which 26 were paid by the government.

In 2003 the Ethiopian government started the implementation of the Health Extension Programme to increase primary health care coverage on the community level. This is the basic level of health care in Ethiopia, consisting of one health post and two associated health extension workers. On average, one health post serves a *Kebele* (county) of 5000 inhabitants. The goals of this programme include improving access to key preventive and curative health services for everyone, with a special focus on maternal and child health [16].

According to the WHO's African Health Observatory, the under-five mortality rate in Ethiopia decreased from 198 to 77 deaths per 1000 live births from 1990 to 2011, corresponding to an annual rate of reduction of 4.5 per cent [17]. Over the same period improvements are also seen for key services such as coverage of measles vaccinations for one-year-olds, increasing from 38 per cent to 57 per cent. However, coverage of key services in Ethiopia remains among the lowest in Africa, as seen in skilled birth attendance, where Ethiopia has the lowest coverage of all the countries included in the African Health Observatory.

Child health outcomes and access to essential maternal and child health services are not equally distributed across all parts of the Ethiopian population. Studies have shown regional differences in coverage for maternal and child health services, and the services are more likely to be used by mothers with formal education, those living in urban areas and the richer parts of the population [18]–[21]. Hosseinpoor et al. found substantial wealth-related inequalities in coverage for several maternal and child health services in Ethiopia [22], and a study on the trends and determinants of neonatal mortality in Ethiopia finds, among other factors, the mother's level of education and region of residence to be associated with the probability of survival [23].

To develop policies aiming at reducing inequality in health outcomes and access to key services, it is important for policy makers to better understand the existing inequalities. There is limited evidence on the combined trends in level and distribution of child health and the underlying factors, that is, information that is necessary to address these challenges in a national context. The objective of this study is to describe the combined level and distribution of coverage for key child health services and outcomes in Ethiopia, and to analyse their association with socioeconomic and geographic determinants.

## Methods

### Data and variables

Data were obtained from the Ethiopian Demographic and Health Surveys (DHS) conducted in 2000, 2005 and 2011 [24]–[26]. These surveys are nationally representative, with sample sizes of 14072, 13721 and 16702 households respectively. For the respective surveys, information was collected on 10873, 9861 and 11654 children. Six different indicators capturing health outcomes and preventive and curative key services were selected for the analysis: Under-five deaths, neonatal deaths, coverage of skilled birth attendance, coverage of basic vaccinations, coverage of oral rehydration therapy for diarrhoea and coverage of antibiotics for suspected pneumonia. Under-five and neonatal deaths are defined as the proportion of live born children who die before the age of five years and four weeks respectively. Skilled birth attendance is defined as the proportion of women reporting that they were assisted by a doctor or a nurse during delivery. Vaccination coverage is defined as the proportion of children aged 12 to 23 months who, at the time of the survey, had received the following vaccines: three doses of DPT, three doses of polio, BCG and measles. Treatment for diarrhoea and suspected pneumonia is defined as the proportion of those reporting symptoms in the past two weeks who have been given oral rehydration therapy (oral rehydration solution or recommended home solution) and antibiotics respectively. The data from the DHS are complimented by the 2007 Population and Housing Census of Ethiopia to obtain population data [27].

### Geographic inequality

The degree of geographic inequality was measured for each of the health indicators in 2011 by what we call the geographic Gini index. The Gini index is closely related to the Lorenz curve, which plots the cumulative proportion of the outcome variable against the cumulative proportion of people ranked by the outcome. The Gini index is defined as twice the area between the Lorenz curve and the diagonal line, called the “line of equality”. The geographic Gini index was calculated for each of the indicators in 2011 based on each region ranked from worst to best achievement of the indicator, and weighed by population size, using the following formula [28]:
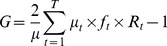
(1)


Where µ is the mean of the health variable in the entire population, T the number of groups, µ_t_ the mean of the health variable in the t^th^ region ant f_t_ its population share. R_t_ is the relative rank of the t^th^ region ranked by the health variable.

### Socioeconomic inequality

The degree of socioeconomic inequality for each of the indicators in 2011 was quantified by the concentration index. The concentration index is analogous to the Gini index, but uses a measure of socioeconomic status for ranking the observations. The concentration index is related to the concentration curve, which plots the cumulative proportion of the outcome variable against the cumulative proportion of the population ranked by a measure of socioeconomic status [28], [29]. The DHS does not contain data on household income or consumption, but the dataset contains a wealth index which was used as a measure of socioeconomic position. This index is calculated by principal component analysis, based on information on household assets (table, radio, refrigerator etc.) and household characteristics (building material, source of drinking water, toilet facilities etc.) [30].

The concentration index equals twice the area between the concentration curve and the line of equality, and for a health variable y it can be expressed as follows [28]:



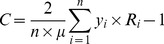
(2)Where C denotes the concentration index, n is the number of observations, µ is the mean of the health variable y, and R is the fractional rank of the individuals by the household's socioeconomic status. The concentration index takes a value between −1 and 1. By convention, the concentration index will take a positive value if the variable in question is more prevalent among the rich, and conversely, a negative value if the variable is more prevalent among the poor. If there is no socioeconomic inequality, the concentration index will take the value 0.

### Decomposition of the concentration index

The concentration indices of the health indicators in 2011 were decomposed in order to determine the contribution of different factors to the overall socioeconomic inequality. The factors included in the decomposition analysis were: mother's education, region of residence, and household's wealth. The factors were chosen on the basis of the conceptual framework used by the WHO and the Commission on Social Determinants of Health [31]. Education was included as a continuous variable, corresponding to the numbers of years of schooling the respondent reported having completed, ranging from zero to eight years. The household's wealth was included as a continuous variable, using the wealth index estimated by the DHS. The 11 regions were included as binary variables in the analysis, with one region chosen as reference on the basis of progress towards the United Nations fourth Millennium Development Goal. The United Nation's fourth Millennium Development Goal calls for a reduction of the under-five mortality by two thirds from 1990 to 2015, which for Ethiopia signifies a reduction from 204 to 68 deaths per 1000 live births over this period. The capital region, Addis Ababa, had already reached this goal in 2011 with 53 deaths per 1000 live births according to the estimates done by the DHS [26], but as this region is not representative for the country, it was not chosen as reference region. The region, apart from Addis Ababa, that is closest to achieving the Millennium Goal is Tigray, with an under-five mortality rate of 85 per 1000 live births. The Tigray region was therefore selected as a reference region for the analysis.

The decomposition of the concentration index has been explained in detail elsewhere [29], [32]. In summary, a decomposition of the concentration index links the different indicators of child health to a set of K determinants, x_1_, …, x_k_, by linear regression:



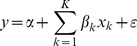
(3)Where y is the indicator in question and ε is an error term. Given the relationship between y_i_ and x_ki_ in equation (3), we get:



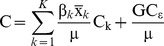
(4)Where C is the concentration index, β_k_ is the regression coefficient in equation (3), is the mean of the determinant k, µ is the mean of the outcome variable y and C_k_ is the concentration index of the determinant k. The last term is the unexplained part calculated as a residual, where GCε is the cumulative concentration index of the error term. Equation (4) is basically made up of two components, the explained component giving the contribution of each determinant, and an unexplained component or residual.

However, this method is developed for continuous outcomes where linear regression is appropriate, and does not allow for binary outcome variables that require non-linear regression models. Van Doorslaer et al. proposed a modification of the standard decomposition method for use in non-linear situations [33]. They propose a probit regression followed by estimation of the marginal effects for each of the explanatory variables evaluated at the sample's mean. The marginal effects go into equation (4) instead of the regression coefficients β_k_ and the linearity required for the decomposition is re-established. The modification proposed by Van Doorslaer et al. was used for the decomposition analysis of all the indicators in this study.

### The health achievement index

The mean level of the indicator and the distributional pattern of the indicator, as estimated by either the concentration index or the geographic Gini index, can be combined into an index of health achievement. The health achievement index was calculated for the socioeconomic distribution of all indicators in 2000, 2005 and 2011, using the following formula [34]:



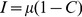
(5)Where I is the health achievement, µ is the mean of the health variable and C it's concentration index.

### Time trends

The mean level, concentration index and health achievement index were estimated for all indicators in 2000, 2005 and 2011. The change in the mean level was assessed by logistic regression, with the indicator in question as dependent variable and the time of the surveys as independent variable.

All statistical analysis was performed using STATA IC version 12.0, taking the sample design into account.

## Results

### Descriptive statistics

Summary statistics of the 2011 indicators as well as a breakdown by maternal and household characteristics is provided in **Table 1**. The neonatal and under-five mortality, as reported by the DHS, was 37 and 88 per 1000 live born children respectively [26]. The proportion of women giving birth assisted by a skilled birth attendant was 10 per cent. 24 per cent of the children aged 12–23 months at the time of the survey had received all basic vaccinations. 30 per cent of the children who reported cases of diarrhoea in the two weeks preceding the survey had been given oral rehydration therapy, and 11 per cent of the children with suspected pneumonia in the two weeks preceding the survey had received antibiotics. The level of coverage and mortality differed according to wealth quintile, level of mother's education and region of residence.

**Table 1:** Summary statistics of the indicators (2011).

	Under-five mortality*	Neonatal mortality*	Skilled birth attendance	Vaccination	Treatment for diarrhoea	Treatment for pneumonia	Number of children
**Wealth quintile**							
Poorest	137	50	1.7%	16.8%	21.7%	7.1%	3625
Poorer	121	48	2.9%	18.2%	24.5%	14.7%	2114
Middle	96	35	3.2%	18.2%	31.7%	7.6%	1872
Richer	100	39	7.4%	24.9%	31.2%	14.2%	1870
Richest	86	37	45.6%	50.5%	50.9%	15.8%	2173
**Mother's education**							
No education	121	46	4.6%	20.1%	25.9%	10.7%	8142
Primary	88	35	15.3%	28.3%	36.1%	11.2%	2930
Secondary	46	31	72.0%	57.0%	57.5%	**	386
Higher	24	8	74.0%	57.7%	**	**	196
**Region**							
Tigray	85	44	11.6%	58.9%	37.1%	7.8%	1202
Affar	127	33	7.1%	8.6%	40.0%	5.8%	1130
Amhara	108	54	10.1%	26.3%	30.4%	9.8%	1294
Oromiya	112	40	8.1%	15.6%	26.1%	11.4%	1761
Somali	122	34	8.2%	16.6%	35.1%	4.8%	1027
Benishangul-Gumuz	169	62	8.9%	23.6%	38.5%	12.7%	1020
SNNP	116	38	6.1%	24.1%	29.0%	14.3%	1614
Gambela	123	39	27.0%	15.5%	48.7%	16.1%	851
Harari	94	35	32.5%	34.1%	44.9%	**	659
Addis Ababa	53	21	83.9%	78.7%	54.4%	**	400
Dire Dawa	97	30	40.3%	58.6%	47.8%	5.1%	696
**National average**	**88**	**37**	**10.0%**	**24.3%**	**29.9%**	**11.0%**	**11654**
**Concentration index**	**−0.12**	**−0.10**	**0.65**	**0.23**	**0.14**	**0.10**	
95% CI	(−0.17)–(−0.064)	(−0.18)–(−0.022)	0.60–0.70	0.15–0.30	0.081–0.20	(−0.065) −0.27	
**Geographic Gini index**	**0.047**	**0.092**	**0.33**	**0.29**	**0.091**	**0.14**	
95% CI	(−0.0068) −0.10	0.029–0.16	(−0.043) −0.70	0.11–0.47	0.026–0.16	0.030–0.25	

*Estimates from the Ethiopia DHS 2011 Final Report [26]. The mortality rates are reported as number of deaths per thousand live births.

**The figure is based on fewer than 25 cases and has been supressed.

### Geographic inequality

**Figure 1:**
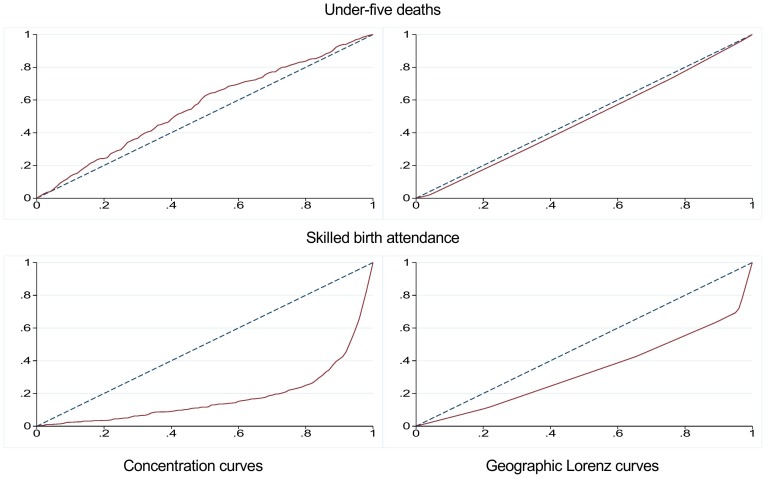
Concentration curves and geographic Lorenz curves for under-five deaths and skilled birth attendance in 2011. The two figures to the left are concentration curves, with the cumulative proportion of the individuals ranked by wealth on the x-axis and the cumulative proportion of the outcome variable on the y-axis. The two figures to the right are geographic Lorenz curves, with the cumulative proportion of the regions ranked from worst to best achievement of the indicator, and weighed by population size on the x-axis. The cumulative proportion of the outcome variable is on the y-axis. The solid red lines represent the concentration and Lorenz curves, and the dashed blue lines represent the “line of equality”.

The geographic Gini indices estimating the inequality between the regions can be found in **Table 1**. The geographic Gini indices ranged from 0.047 (95 per cent confidence interval ((−0.0068) −0.10) for under-five deaths to 0.33 ((−0.043) −0.70) for skilled birth attendance. **Figure 1** displays the geographic Lorenz curve for under-five deaths and skilled birth attendance.

### Socioeconomic inequality

The concentration indices for each of the indicators in 2011 can be found in **Table 1**. For all indicators except treatment for suspected pneumonia, the concentration indices were significantly different from zero at a 95 per cent significance level. The absolute values of the concentration indices were above or equal to 0.10 for all indicators. The lowest degree of socioeconomic inequality was found for neonatal deaths and antibiotics, with concentration indices of −0.10 ((−0.18)–(−0.022)) and 0.10 ((−0.065) −0.27) respectively. The indicator revealing the largest degree of socioeconomic inequality was skilled birth attendance, with a concentration index of 0.65 (0.60–0.70). For under-five and neonatal deaths, the concentration indices were negative, indicating that a disproportionate fraction of these deaths occurs in children of poor families. The concentration indices for coverage of skilled birth attendance, vaccinations, oral rehydration therapy for diarrhoea and antibiotics for suspected pneumonia were positive; these services were therefore more prevalent among the wealthier part of the population. **Figure 1** displays the concentration curve for under-five deaths and skilled birth attendance.

### Decomposition analysis

**Figure 2:**
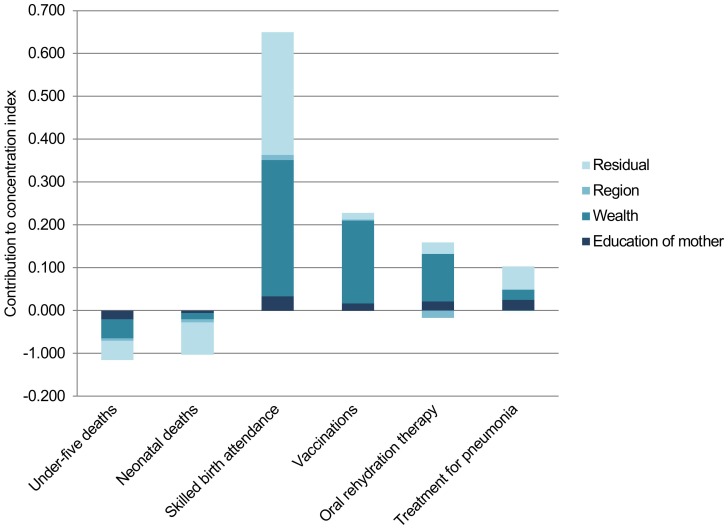
Factors contributing to socioeconomic inequality. Contribution of wealth, the mother's education and region of residence to the total socioeconomic inequality, as measured by the concentration index, for each of the indicators in 2011.

The results of the decomposition of the indicators' concentration indices in 2011 are presented in **Table 2** and graphically in **Figure 2**. The wealth factor alone accounts for the majority of the explained inequalities for all indicators, with a contribution ranging from 13.6 per cent of the total inequality in neonatal deaths to 84.8 per cent for coverage of basic vaccinations. The percentage contribution of wealth in the decomposition analysis is an estimate of the pure effect of wealth on the total inequality, adjusting for other relevant factors. Education accounts for a smaller proportion of the inequalities, with a contribution ranging from 5.1 per cent of the total inequality in skilled birth attendance to 24.6 per cent in treatment of suspected pneumonia. The proportion of the inequalities not explained by systematic variations in the explanatory variables is captured by the residual, the lowest is found for coverage of vaccinations where 6.4 per cent of the inequality is not explained by the model, and the highest is found for neonatal deaths with a residual of 73.8 per cent.

**Table 2:** Absolute and percentage contribution of mother's education. wealth and region of residence to the concentration indices (C): results of the decomposition analysis.

	Under-five deaths		Neonatal deaths		Skilled birth attendance		Vaccination		ORS		Antibiotics	
	Absolute	%	Absolute	%	Absolute	%	Absolute	%	Absolute	%	Absolute	%
**Education of mother**	−0.020	17.5	−0.006	5.7	0.033	5.1	0.017	7.4	0.022	15.3	0.025	24.6
**Wealth**	−0.045	39.0	−0.014	13.6	0.318	48.9	0.193	84.8	0.111	77.6	0.024	23.5
**Region**												
Tigray	Ref.	Ref.	Ref.	Ref.	Ref.	Ref.	Ref.	Ref.	Ref.	Ref.	Ref.	Ref.
Affar	0.000	0.4	0.003	−2.6	0.001	0.1	0.006	2.6	0.000	−0.2	0.001	0.6
Amhara	0.001	−0.5	−0.001	0.8	−0.004	−0.6	0.017	7.6	0.002	1.3	−0.001	−1.2
Oromiya	0.000	0.0	−0.003	2.7	0.002	0.3	−0.027	−12.0	−0.013	−9.3	0.006	5.8
Somali	0.000	0.0	0.002	−1.6	0.001	0.1	0.004	1.5	0.000	−0.3	0.003	3.3
Benishangul-Gumuz	0.000	0.3	0.000	0.2	−0.001	−0.1	0.001	0.4	0.000	−0.2	0.001	0.5
SNNP	−0.001	0.8	0.002	−1.5	0.001	0.2	0.015	6.7	0.002	1.5	−0.002	−1.7
Gambela	0.000	0.1	0.000	0.0	0.000	−0.1	0.001	0.4	0.000	−0.2	−0.001	−0.9
Harari	0.000	−0.1	0.000	0.0	0.001	0.1	−0.001	−0.7	0.000	0.0	*	*
Addis Ababa	−0.004	3.6	−0.008	8.2	0.011	1.6	−0.012	−5.3	−0.006	−4.5	−0.007	−7.1
Dire Dawa	0.000	0.2	−0.001	0.9	0.001	0.1	0.000	−0.01	0.000	−0.002	0.000	−0.1
**Residual**	−0.045	38.8	−0.076	73.8	0.287	44.1	0.015	6.4	0.027	18.9	0.054	52.6
**Total**	**−0.116**	**100.0**	**−0.103**	**100.0**	**0.650**	**100.0**	**0.228**	**100.0**	**0.142**	**100.0**	**0.102**	**100.0**

*The contribution of the region Harari to the inequality in use of antibiotics was omitted in the regression analysis because ALL = 0.

### Time trends

**Figure 3:**
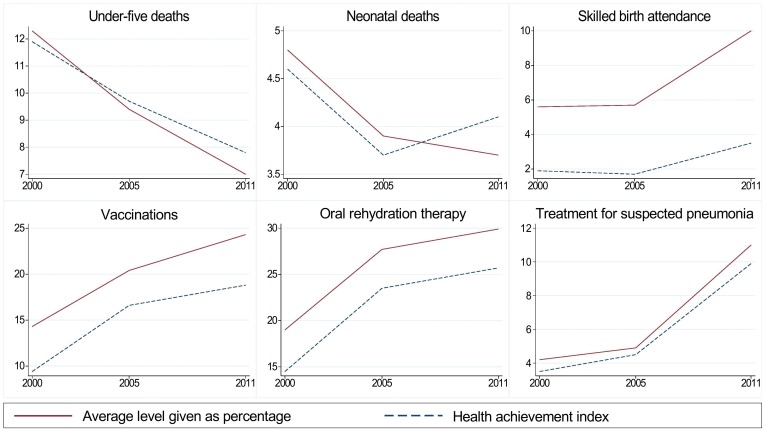
Changes in average level (given as percentage) and health achievement index from 2000 to 2011. Change from 2000 to 2011 in average level and health achievement index for each of the indicators. The time is on the x-axis, and the health achievement index and average level or coverage are on the y-axis.

The mean level, concentration indices and health achievement index for all variables in 2000, 2005 and 2011 can be found in **Table 3**. Since geographic inequality as measured here accounts for very little of the explained inequalities, we did not include a geographic achievement index. The mean of all indicators improved from 2000 to 2011. The concentration indices revealed increasing socioeconomic inequalities for under-five and neonatal deaths, and somewhat decreasing or unchanged inequalities for the remaining indicators. The health achievement index shows an improvement for all indicators except neonatal deaths. The change over time in mean level and health achievement is shown graphically in **Figure 3**.

**Table 3:** Change in average level and health achievement index from 2000 to 2011.

	Average/coverage	Concentration index (95% CI)	Health achievement index	Average/coverage	Concentration index (95% CI)	Health achievement index
	**Under-five deaths**			**Neonatal deaths**		
**2000**	12.3%	0.029 ((−0.010) −0.067)	11.9%	4.8%	0.037 ((−0.028) −0.10)	4.6%
**2005**	9.4%	−0.030 ((−0.071) −0.012)	9.7%	3.9%	0.052 ((−0.016) −0.13)	3.7%
**2011**	7.0%	−0.12 ((−0.17)–(−0.064))	7.8%	3.7%	−0.10 ((−0.18)–(−0.022))	4.1%
**p for trend**	<0.001			0.008		
	**Skilled birth attendance**			**Oral rehydration therapy**		
**2000**	5.6%	0.66 (0.61–0.72)	1.9%	19.0%	0.24 (0.16–0.31)	14.5%
**2005**	5.7%	0.70 (0.65–0.75)	1.7%	27.7%	0.15 (0.082–0.22)	23.5%
**2011**	10.0%	0.65 (0.60–0.70)	3.5%	29.9%	0.14 (0.081–0.20)	25.7%
**p for trend**	<0.001			<0.001		
	**Vaccination**			**Treatment for suspected pneumonia**		
**2000**	14.3%	0.34 (0.26–0.43)	9.4%	4.2%	0.17 (0.037–0.30)	3.5%
**2005**	20.4%	0.18 (0.10–0.27)	16.6%	4.9%	0.088 (0.088–0.088)	4.5%
**2011**	24.3%	0.23 (0.15–0.30)	18.8%	11.0%	0.10 ((−0.065) −0.27)	9.9%
**p for trend**	<0.001			<0.001		

## Discussion

This study demonstrates the presence of geographic inequalities and pro-rich inequalities for all indicators in 2011. The major contributor to the observed socioeconomic inequality in access to key services and health outcomes is wealth. The mean level of all indicators improved from 2000 to 2011. Socioeconomic inequalities seem to decrease for most but not all indicators from 2000 to 2011, while the health achievement index shows improvement for all the indicators except neonatal deaths.

Other studies have found mother's educational level, wealth and region of residence to be important determinants for child health and access to health services in Ethiopia [21]–[23], which is in line with this study. However, in this study a combination of different methods for assessing inequalities is used, which leads to a better and more nuanced understanding of the current situation and changes over time. Quantifying inequalities using the concentration index provides a useful tool for comparing the magnitude of the inequalities for different services, and for assessing the changes in inequality over time. The health achievement index, that incorporates socioeconomic inequality and the average level in the population into one metric, gives useful additional evidence to policymakers concerned with both of these aspects.

Previous studies decomposing socioeconomic inequalities in child mortality and skilled birth attendance in low and middle income countries have found that the mother's education and wealth are the main contributors to overall socioeconomic inequalities [1], [3], [35]. The proportion of total inequality that is attributable to education is higher in these studies than the results of our study indicate. This may be explained by the low coverage of key services in Ethiopia. Few people have access to the services; it is therefore not unexpected that there are large disparities in access across the population and that wealth is the most important determinant for accessing key services. A review comparing inequalities in several low and middle income countries finds that the coverage in the lower wealth quintiles are subject to more variability than coverage in the richest quintile [4], suggesting that the richest part of the population have the means to receive needed services irrespective of how the country's health system is functioning.

The lowest degree of socioeconomic inequality is found for neonatal deaths and treatment for suspected pneumonia. Wealth contributes to a comparatively smaller degree of the socioeconomic inequality for neonatal deaths than for the other indicator, and a large proportion of the inequalities is not explained by the decomposition. This might be due to the large impact of biological factors, health system factors and other factors that are not included in our model.

Access to antibiotics for pneumonia was included as a binary variable indicating whether children presenting symptoms in the two weeks preceding the survey had received antibiotics. The weakness of this classification is that those who seek medical care, but for whom antibiotics are not needed, are classified as not having access to treatment. However, the alternative indicator measuring access to treatment of pneumonia by whether medical advice was sought, will not take account of the quality of the consultation or the availability of drugs.

When decomposing inequalities in health outcomes and access to key services, the geographic determinants account for a relatively small proportion of the inequalities. The geographic determinants are included as regions, and one possible explanation of the relatively small contribution of the regions may be that the major part of the geographic inequality is due to factors on a more detailed level than the regional level that we measure, for example, walking distance to the closest health facility. A study from a rural area in north-western Ethiopia found that children who had more than one and a half hour travel time to the nearest health centre had a two- to threefold greater risk of dying before the age of five than children living within one and a half hour from the health centre [7]. A study from Burkina Faso reports similar findings [8].

Assessing whether the situation is improving for each of the indicators depends on the measure used. If one is only concerned with the mean level in the population, there has been a positive evolution for all indicators from 2000 to 2011. If one is only concerned with the distribution across socioeconomic groups, the results are more diverse, indicating increased inequality for some indicators and reduced inequality for others. This has been shown for several other countries as well [12], [14]. However, we argue that it is crucial to achieve both a higher mean level and more fairly distributed health and coverage of key services. The health achievement index is a way of incorporating both of these concerns into one metric. The health achievement index improved for all indicators from 2000 to 2011, except neonatal deaths. This means that even where the concentration index is worsening, the increase in inequality is outweighed by improvement of the mean level. To date, few studies have combined the information available on coverage and on distribution into a single metric such as the health achievement index. A study from Nigeria uses the health achievement index to assess malnutrition, and emphasises the importance of including both inequality and the mean level, because subgroups of the population that do well in one dimension often do less well in the other dimension [36]. A study that uses the health achievement index to assess time trends in measles vaccination in 21 low and middle income countries finds both increasing and decreasing health achievement indices [13].

The changes in mean level, inequality and health achievement are assessed from 2000 to 2011 in our study. This corresponds to the time period when the Health Extension Programme was implemented [16]. The Health Extension Programme focuses on community based services, with the aim of improving health outcomes and coverage of key services and making key services universally accessible. Studies evaluating the impact of the Health Extension Programme in Ethiopia find that the programme has contributed to increased coverage of vaccinations, improved maternal and neonatal health care practices and improvement of health-promoting and care-seeking behaviour, but the programme does not seem to have impacted coverage of skilled birth attendance and postnatal care [37]–[40]. Our study has not assessed the effects of the Health Extension Programme, but the results of our study should be seen in relation to the implementation of the Health Extension Programme.

This study is based on data from the Demographic and Health Surveys. These surveys are conducted in many low and middle income countries with standardised questionnaires. The surveys are nationally representative with a relatively large sample size. However, the estimates done by the Ethiopian Ministry of Health differ somewhat from the DHS' estimates for some indicators. For example, the 2011 report on health and health related indicators published by the Ethiopian Ministry of Health estimates measles coverage to be 82 per cent [41], whereas it is estimated at 56 per cent by the 2011 Ethiopian DHS [26].

There are several limitations to this study. First, the decomposition analysis is based on regression analysis with varying degree of statistical significance. The results of the decomposition analysis should therefore be interpreted with caution. Second, factors other than those incorporated into the models may exclude people from receiving health care. Supply side factors, such as the presence of health facilities, quality of care and the availability of drugs may be important reasons why people are not receiving needed health care. This is not fully accounted for in the model, although it is partly explained through the geographic inequalities. Demand side factors, such as cultural barriers, costs of receiving health care and time available to seek medical care, are not explicitly incorporated into the model due to data limitations.

## Conclusion

Socioeconomic and geographic inequalities exist in the distribution of access to key services and health outcomes in Ethiopia. Wealth is the major determinant of socioeconomic inequality in child health, and there are widening inequalities for some of the indicators included in this study. However, the mean level of health outcomes and coverage of key services is improving, and the health achievement indices show improvements for all indicators with the exception of neonatal deaths.
